# Supplementary material for: Patterns of Intron Gain and Loss in Fungi
Source: PLoS Biol. 2004 Nov 30;2(12):e422. doi: 10.1371/journal.pbio.0020422 (PMC532390; doi:10.1371/journal.pbio.0020422)
Supplement: Table S1 — Also available at http://genes.mit.edu/NielsenEtAl/. (4.3 MB ZIP). [file pbio.0020422.st001.zip › NielsenEtAl/html/533.html]

AN7302.1.NCU00162.1.MG05685.1.FG02529.1


```
 CLUSTAL W (1.82) Multiple Sequence Alignments - Introns Inserted


Sequence 1: NCU00162.1	436 aa
Sequence 2: MG05685.1	450 aa
Sequence 3: FG02529.1	445 aa
Sequence 4: AN7302.1	419 aa
Alignment Length: 457 aa
Number Identitical Residues: 105 aa
Alignment Score (without introns) 6759


MG05685.1 	MHFRSSLAWSMVASATQVQAQYLLNDMSFGVGSK2IS-ENG-HTIPNFSTQGNPRLPEL~
NCU00162.1	---MRLLVGIVALGSVAEAAQYLVSDLSFGFGAR2IN-PDGRNTIPNYSMQGNPNVPEL~
FG02529.1 	--MKVSFSLAALLAASLSQAQYLISELSFGHAGR2ISPAESRGQIPNFAVQGNPNTPEI~
AN7302.1  	----MKVPALFFFFSAVATAQTVIESSSFGHGAM2VA--PNREGIPGWDIGGEGHFPQI0
          	      .       :    ** ::.. *** ..  :    .   **.:   *: . *:: 

MG05685.1 	LSNKLILTPPAPGNQRGAVWTDKE~LHQDEWVTDVSFRVNGPERGGGNMNIWMVRDGSRE
NCU00162.1	LSNKVILTPPAPGNQRGAVWADKP~LEHRAWTTDIDFRANGPERGSGLLNIWLAKDGARS
FG02529.1 	LSNRIILTPLAPGNQRGAIWGQQP~LLRTQWIADVDFRANGPDRGRGNLNIWLVRNGPAT
AN7302.1  	LSNKVILTPPYPGNTRGYAWSQTP2LSQSEWAAEFQFRATGVERAGGNLQLWYAKDGKQK
          	***::****  *** **  * :   * :  * ::..**..* :*. * :::* .::*   

MG05685.1 	VGTSSIYTVGHFEGLALVVDTYGGS~GGMIRGFLNDGSTDY-RSAHVDGLAFAHCNYAYR
NCU00162.1	IGSQSIYTVGKFEGLVLVVNQHGGS~AGMIRGFLNDGTLDYSKRDNVDNLAFGHCWFAYR
FG02529.1 	IGAGSIYTVGKFDGLALVIDTSGGS~SGMVRGFLNDGNTDYSRQNNVDELSFGHCPYNYR
AN7302.1  	IGSASIYTVGQWDGFALVVDTHSGR0GGSIRGFLNDGTTDYKSHSSVDSLAFGHCDYSYR
          	:*: ******:::*:.**::  .*  .* :*******. **.    ** *:*.** : **

MG05685.1 	NLGRPSEIKVTQTQHNFRVDVDSSLCFESTT~VGIPGGYKVGVTAASADNPDSVELFKVA
NCU00162.1	NLGRPSQIKLRHNSQNFKVEVDGRLCFESDK~IHIPTGYNFGLSAASAENPDSFEVFKVV
FG02529.1 	NLGRPSQVKLRQTARAFRVELDGKLCFESDK~ISIPTGYQFGVTAATPDNPDSFEVFKMV
AN7302.1  	NLGRPSVIRVRSTNAIFEVTVDDKPCFATDK0ITLPAGNTIGVTAATPENPDSFEVFKVV
          	****** :::  .   *.* :*.  ** : . : :* *  .*::**:.:****.*:**:.

MG05685.1 	VLSHQVGKTQDHHDDHHDAGHDNSHAHDDHGAEHDNEKAHTPSKFSRGHSEPEHGSSHAE
NCU00162.1	VLS---------EDDIGTVPNQQQQQQQQQSGENQKPLGGT----NHGRSGQ---AAIQD
FG02529.1 	VMAD--------NSDTGAVRNDPPKQQNQNQNQNQNQNQNQNQNQARGQPAKANSNNNNN
AN7302.1  	LQT-----------ATSPVGKAPPVQAQN---TNQQPLADQ----PQGNARQEQGFSNN-
          	: :               . :      ::    :::          :*..    .     

MG05685.1 	HWDEDIPDESASKYTSSKAQFEDLHNRLQSVSHHLSTIFKQVTAQHSMDEHRHEETSSMI
NCU00162.1	PYDNAIPDAEANSITSSKAQFEDLHTRIQSLNHHLSSIFRSVAQYGGIADQNHQEVSIML
FG02529.1 	RNDGEYADEDPDIFSTSKTQFMDLHNRLQNTNHQLSSLQRTSSRHQQQDEKRHEDLTSLI
AN7302.1  	-------------LNSINAQFVDLGSRIQLVNHATNNIIRELGNQASKSETRQLELLQRV
          	              .: ::** ** .*:*  .*  ..: :         : .: :    :

MG05685.1 	NDVK---TALGKLHDIDSRMHSLEQEVRSIR-ADLSHKVQTSENNIRTFVGDTHFKTVQT
NCU00162.1	GEMKGLLTKLDRFASVENKLENLEREVRSMR-SELTARLKESEHSIKYHVTDKHEDLAGH
FG02529.1 	GQLRADMRKLDDITALNSRISELEKDIQGLR-KELTRKLQSTERTFKTTLSDHHSTLS-E
AN7302.1  	ATKEQVQSLEARLQRMEQTLLSIQRDLEGKDYRDRFNQLHETLRSSHQSLTENLKDNVVS
          	   .        :  ::. : .:::::..    :   ::: : .. :  : :        

MG05685.1 	V~QKHAAPGHSRLIFVIIASQLLLVVGYGVYKRRKASMPKKYL
NCU00162.1	V~VKHAAPGHTKLYLAIVGSQVVLAAAYFLYKRRKAGGPKKYL
FG02529.1 	V~VMNKTPGHKFLIFSIIGTQCILVAAYVVYKRRRASMPKKYL
AN7302.1  	V1ITASTPRMGFFIFSMIAFQVFLAVSYIIYKRRRANMPKKFL
          	*     :*    : : ::. * .*...* :****:*. ***:*
```
